# A Closed-Loop Approach to Fight Coronavirus: Early Detection and Subsequent Treatment

**DOI:** 10.3390/bios12100900

**Published:** 2022-10-20

**Authors:** Guoguang Rong, Yuqiao Zheng, Xi Yang, Kangjian Bao, Fen Xia, Huihui Ren, Sumin Bian, Lan Li, Bowen Zhu, Mohamad Sawan

**Affiliations:** 1 CenBRAIN Neurotech, School of Engineering, Westlake University, 600 Dunyu Road, Xihu District, Hangzhou 310030, China; 2 School of Engineering, Westlake University, 600 Dunyu Road, Xihu District, Hangzhou 310030, China; 3 Institute of Advanced Study, Westlake Institute for Advanced Study, Hangzhou 310024, China

**Keywords:** biosensing, coronavirus, SARS-CoV-2, COVID-19, early diagnosis, epidemic control, respiration monitor, photoacoustic imaging, neurostimulation

## Abstract

The recent COVID-19 pandemic has caused tremendous damage to the social economy and people’s health. Some major issues fighting COVID-19 include early and accurate diagnosis and the shortage of ventilator machines for critical patients. In this manuscript, we describe a novel solution to deal with COVID-19: portable biosensing and wearable photoacoustic imaging for early and accurate diagnosis of infection and magnetic neuromodulation or minimally invasive electrical stimulation to replace traditional ventilation. The solution is a closed-loop system in that the three modules are integrated together and form a loop to cover all-phase strategies for fighting COVID-19. The proposed technique can guarantee ubiquitous and onsite detection, and an electrical hypoglossal stimulator can be more effective in helping severe patients and reducing complications caused by ventilators.

## 1. Introduction

Since December 2019, there has been a large outbreak of COVID-19 pneumonia worldwide, which has greatly impacted economic development and social operation [1]. This new coronavirus pneumonia is caused by an infection of the SARS-CoV-2 virus and is an acute respiratory infection. An important reason for the outbreak of the COVID-19 epidemic is that the current detection methods are slow and inefficient. In the process of detection and treatment, large and medium-sized medical devices and equipment are involved. A large number of patients are likely to cause the collapse of the existing medical system. The main medical instruments used in the existing COVID-19 detection and treatment stage, including the Polymerase Chain Reaction (PCR) test and the Computed Tomography (CT) scanning instrument and ventilator, require a long evaluation time. Most of the corresponding equipment is bulky and expensive and requires a professional operation, making it difficult to apply on a large scale. Therefore, a portable and easy-to-operate detection platform with an automatic data-processing system running the algorithm is proposed to increase detection and treatment efficiency.

At present, the detection of COVID-19 needs to be completed by medical authorities in clinical conditions with the prevention of the Centers for Disease Control. The main technology is based on a polymerase chain reaction, a kind of nucleic acid amplification test (NAAT) [2]. However, nucleic acid extraction and amplification is a complex process. The accuracy of the results is affected by the experiences of operators, the cleanliness of the environment and the timeliness of sample delivery. Therefore, although the NAAT method, represented by PCR, has high sensitivity and specificity, the successful detection rate is still low, the result turnaround time is long (typically >4 h), and the frequency and population coverage are limited [3]. Although some agencies have proposed COVID-19 onsite detection solutions, such as isothermal NAAT, automatic nucleic acid extraction NAAT, and onsite NAAT in a mobile laboratory, these methods only try to minimize human factors in nucleic acid extraction and amplification or create a relatively clean environment at the detection site to improve reliability. In fact, they still face the problems of long detection cycles and sample pollution caused by sample transmission and cannot achieve rapid detection and fast delivery of results.

These factors limit the application of NAAT detection in the prevention and control of COVID-19. To detect COVID-19 infected people more accurately, a CT scan was used to perform lung imaging in the clinical setting. The lung injury was detected and combined with the results of a clinical PCR and antibody tests to comprehensively diagnose the infection. However, CT can only be completed in hospitals, the Center for Disease Control (CDC), and other institutions, which is difficult to popularize on a large scale. Moreover, a CT scan necessitates a long processing time, the subjects need to queue up in turn, and cross-infection is easy to occur in the process of queuing. In addition, in the early stage of infection, the pathological characteristics of lung tissue are not obvious enough. As such, its role in early population screening is limited [4]. After infection by SARS-CoV-2, patients are prone to symptoms of dyspnea, especially in critical patients. They need a ventilator to help them breathe. However, once patients begin to use the ventilator, they cannot easily leave the ventilator and breathe independently, and some patients may have complications [5].

Given these realities and the need for epidemic prevention and control, it is urgent to achieve rapid, low-cost, onsite detection of COVID-19, especially an efficient way to quickly screen infected persons in a large population. Such technical means can help epidemic prevention personnel quickly implement the corresponding isolation, diagnosis, treatment, and recovery processes and provide a solid technical foundation for epidemic prevention and control and the resumption of social activities. In view of this, we propose an efficient COVID-19 solution scheme covering the whole process of the early, middle, and late stages of disease development, including the technical means of in vitro diagnostic technology, tissue imaging detection technology, breath detection, and electrical stimulation technology. With such a large span and wide coverage, we aim to innovate for all aspects of COVID-19’s influence on the human body as far as possible. Moreover, the technical means adopted in this work are based on the rapid application on site, such as portable or wearable devices. The goal is to facilitate the deployment on complex sites and eliminate the dependence on large instruments and a professional environment.

**Figure 1:**
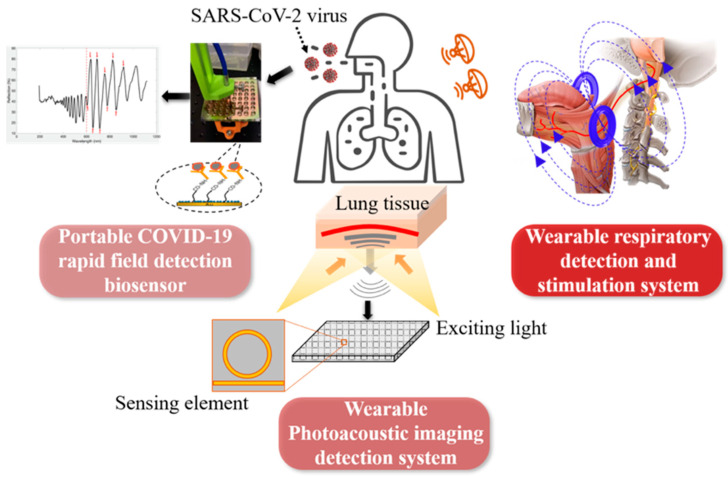
Proposed closed-loop solution for COVID-19, including three main parts: portable biosensor, wearable photoacoustic imaging, and breath detection and magnetic as well as electrical stimulation.

As shown in Figure 1, the three main parts of this project include: (1) A portable COVID-19 rapid field detection biosensor, which can detect trace virus particles from the swab, sputum, saliva, and bronchoalveolar lavage fluid, and directly detect virus infection from the biochemical, microbiological, and in vitro diagnostic level. Meanwhile, compared to the rapid antigen test, the biosensing system has higher sensitivity and a lower limit of detection (around 100 copies/mL vs. 10,000 copies/mL), better specificity, lower cost (<1 $ per test), and higher throughput (>384 tests per half hour) [6]. (2) A wearable nanoimaging detection system based on the photoacoustic imaging method to scan the upper respiratory tract or lung tissue in order to detect the change of blood oxygen content in the tissues or blood vessels invaded by the virus before the onset of symptom. This technology can capture the subtle signs of virus infection from the tissue imaging level. (3) A novel respiration detection and stimulation system first detects respiratory abnormalities caused by COVID-19 infection, then applies electrical/magnetic stimulation to assist breathing difficulties or restore spontaneous breathing to patients who rely on a ventilator. This can detect COVID-19 infection and relieve clinical symptoms.

There are researchers working on optical biosensors [7], photoacoustic imaging [8], respiration monitoring [9], and neurostimulation [10]. However, combining all these aspects together to form a closed-loop approach to fight COVID-19 in all stages of disease development is a novel idea. The above three main directions of this work can complement COVID-19’s early infection, medium-term development, and the whole process of intensive care. The effective combination of these directions will provide powerful tools for epidemic prevention, control, and treatment, and the applications will produce significant social and economic benefits. For example, the combination of portable in vitro diagnosis and wearable photoacoustic imaging can quickly diagnose infected people at an early stage and can be applied to population screening because of its high efficiency and speed. For another example, the combination of wearable photoacoustic imaging and respiratory detection and stimulation system can be applied in moderate and severe patients. It accurately grasps the patient’s condition development in combination with tissue injury and clinical respiratory status and applies electrical stimulation to relieve symptoms before using a ventilator and intubation oxygen delivery, to provide valuable time for clinical treatment.

## 2. Proposed System Modules

### 2.1. Portable Biosensing

**Figure 2:**
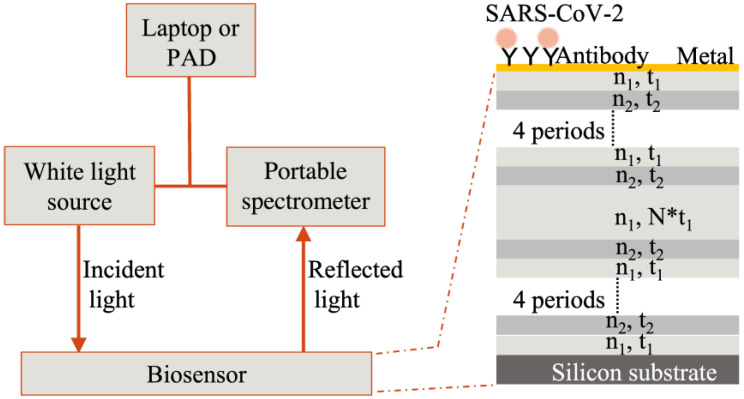
Biosensor and its portable detection system. Two different porous silicon layers construct the resonant microcavity, with an optical refractive index and thickness of n_1_, t_1,_ and n_2_, t_2_. There are two 4-periods structures sandwiching a defect layer with thickness N times t_1_. N can be any number except 1. For details, see reference [11]. Due to the nanoporous structures of porous silicon, a conformally deposited metal thin film is also nanoporous, which allows LSPR excitation by incident light.

Figure 2 shows the schematic diagram of the biosensor and its portable detection system. This sensing device is based on a combination of porous silicon resonant microcavity with localized surface plasmon resonance (LSPR). Porous silicon microcavity includes top and bottom distributed Bragg reflectors (DBR), and a defect layer has the resonant feature of a high-quality factor in its reflection spectrum [11]. The thin metal film conformally deposited on the porous silicon also has nanoporous structures, which allows the excitation of LSPR resonant mode by incident light. LSPR has strong electromagnetic field confinement in the vicinity of the nanoporous metal thin film. The mode coupling between resonant microcavity and LSPR provides high sensitivity toward virus binding on biosensor surfaces [6]. The high-quality resonant feature of the resonant microcavity makes it optimal to resolve small shifts caused by low virus concentration in the specimen.

The detection of the biosensor is based on reflection spectroscopy. A portable and low-cost fiber spectrometer can be used to obtain the reflection spectrum of the biosensor. The biosensor surface has been coated by a specific antibody towards SARS-CoV-2. This latter is a pseudovirus prepared in artificial saliva that was used in this work. When the virus binds with the custom protein or antibody immobilized on the biosensor surface, the refractive index of the physical space occupied by the virus-antibody pair, which is also approximate to the biosensor surface, will increase. This will result in a redshift of the resonant-feature characteristics in the reflection spectrum of the biosensor. This resulting spectrum is measured twice before and after virus binding, and by calculating the amount of quantitative redshift information, the virus in the specimen can be obtained.

### 2.2. Wearable Photoacoustic Imaging

Photoacoustic imaging (PAI) [12] is a non-invasive imaging technique without radiation, which combines the advantages of optical imaging and acoustic imaging. Based on the photoacoustic effect, a modulated pulse light can generate ultrasound signals inside the tissue. And the photoacoustic signals can be received by the ultrasound transducers and reconstructed into images. Using endogenous light-absorbing contracts of biological tissues, PAI can present the anatomical and functional information of different tissues, including hemoglobin, myoglobin, lipids, melanin, water, DNA, RNA, and bilirubin, among others [13], which can be used for disease detection and daily inspection.

**Figure 3:**
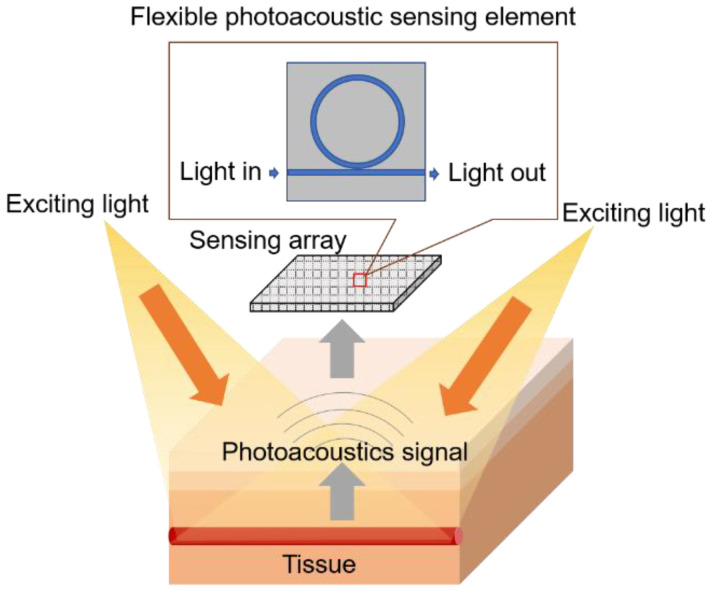
Flexible electronic device for photoacoustic sensing.

The ultrasound sensor is one of the most important parts of a PAI system. Traditional ultrasound transducers detect the photoacoustic signal by converting pressure signals to electrical signals. The transducers have narrow bandwidth and are limited for further miniaturization [14]. The systems need transducer arrays or a scanner to increase the field of view, which restricts the size of the probe and the precision of the detection. Some researchers use integrated photonics to detect photoacoustic signals with sensors of great performance [14,15]. Flexible devices will make transducers better in contact with human bodies, and they are one of the solutions for future wearable devices. Authors are integrating ultrasonic transducers on flexible substrates and using them for human body sensing [16,17]. Figure 3 shows the schematic of flexible electronic systems intended for application in photoacoustic sensing.

### 2.3. Respiration Monitoring and Electrical/Magnetic Stimulation

Unfortunately, patients with COVID-19 may easily be affected by upper airway obstruction [18]. This obstruction is a life-threatening disease that needs consistent assessment and either non-invasive or different invasive intervention methods [19]. Among a series of methods to treat upper airway obstruction, electrical stimulation shows a potential high-performance solution [20]. The principle of an electrical stimulation system is to treat upper airway obstruction since the genioglossal muscle can be contracted when stimulating the hypoglossal nerve. The genioglossal muscle is the largest dilator muscle. With the contraction of this muscle, the upper airway can be enlarged, and obstruction can be relieved and treated.

**Figure 4:**
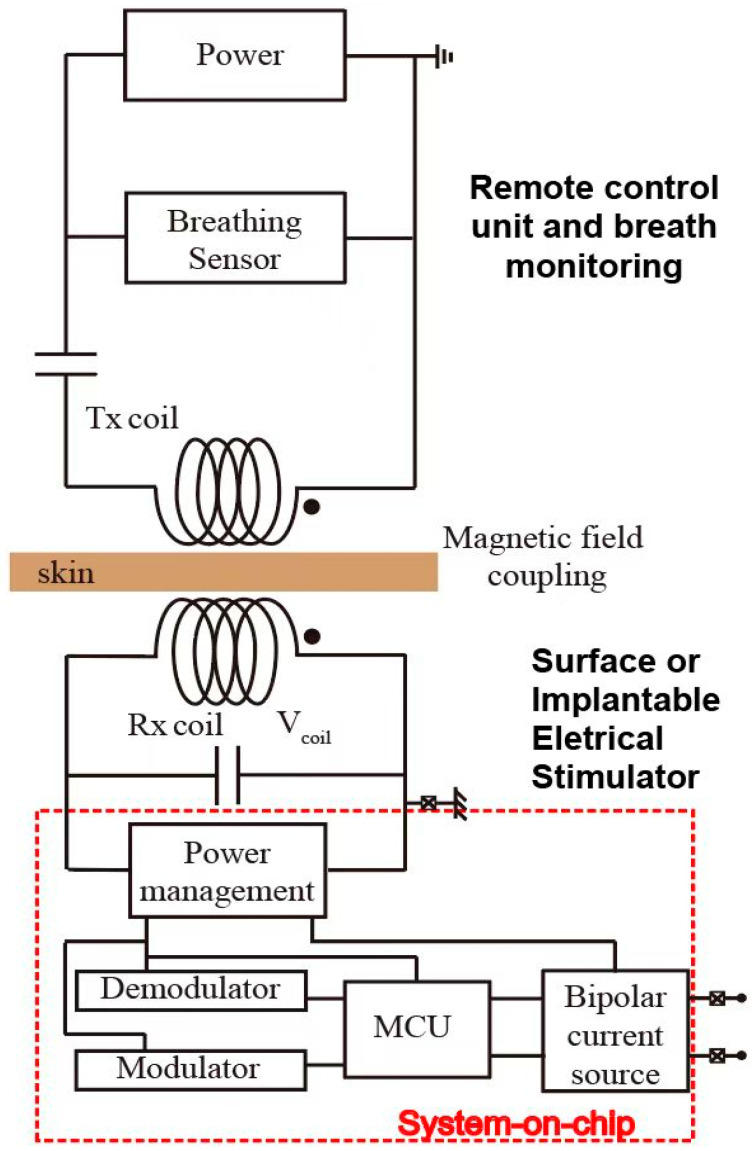
Breathing monitor and subsequent electrical neurostimulation interface.

Figure 4 shows the breathing monitor and electrical stimulation system. This system consists of a respiratory detection unit and an electrical stimulation device. If patients are affected by upper airway obstruction, the respiratory waveform will be different from that of normal people. This respiratory difference can be detected through a pressure sensor. Recently, many detection methods for human breathing have been roughly divided into contact and non-contact respiratory monitoring techniques [21]. Compared to the non-contact respiratory method, the contact approach possesses higher accuracy due to direct contact with the body measurement. Among them, the flexible pressure sensor has the advantages of being small in size and easy to be merged within clothing, which is selected to perform the respiratory monitoring task.

Previous studies have demonstrated the high sensitivity and conformality of our flexible pressure sensor, which lays the groundwork for subsequent respiratory detection [22]. Then, the respiratory monitoring unit transmits this abnormal respiratory signal to the electrical stimulation block to apply the stimulation process. The electrical stimulation block consists of a power management module (rectifier, regulator), modulator, demodulator, control unit, and biphasic current source.

The received electromagnetic voltage signal is converted to DC voltage level through the power management module to supply power to the electrical stimulation module. The regulated DC voltage is used to initiate the stimulation process. The control unit of the electrical stimulation block updates the stimulating parameters to the stimulator’s current source, which is then applied to the hypoglossal nerve after receiving the activation instruction from the respiratory monitoring circuit. Two alternative stimulation approaches are included: (1) Purely magnetic stimulation, which is remotely applied from outside the body. (2) An electrical stimulation device to be implanted under the skin close to the hypoglossal nerve through two-contact electrodes for more critical cases.

## 3. Technical Challenges and Results

Several technical issues must be solved to achieve the goals of the proposed closed loop solution for fighting COVID-19. The most important of them is representative of the major obstacles. Solving these issues will secure research breakthroughs for the proposed system.

### 3.1. Portable Biosensing

It is fully automatic and has high throughput, high sensitivity, and high-accuracy field detection of COVID-19. In view of the characteristics of COVID-19’s rapid transmission and high risk of infection, the study of a high-throughput detection method can achieve large-scale screening. The higher sensitivity can detect infected persons in the early stage to isolate patients and apply the early treatment. The fully automated system can avoid the infection of operators or the cross-contamination between samples.

Expected coronavirus biosensors must be consumables. Their design and integration are intended for mass production potential. If they can be mass-produced, they can be deployed in a large area and realize their potential in onsite detection. The mass production of biosensors also requires reliable and stable quality, which can maintain the validity of the biosensor to facilitate transportation and storage. The silicon wafer-level production of porous silicon has been industrialized, establishing a good foundation for the large-scale production of biosensors. Therefore, the proposed devices in this work are significant for preventing and controlling COVID-19 in a large-scale application.

Considering the nanopore selectivity and surface chemical characteristics of a porous silicon biosensor, the requirements of sample pretreatment should be studied. The treatment of clinical samples is also of great significance for the detection results for the whole detection process. Therefore, the development of pretreatment methods for different samples, such as pharyngeal swab sampling, sputum, or bronchoalveolar lavage fluid, is very important to give full play to biosensor performance. Preliminary studies show universal viral medium (UVM) or universal transport medium (UTM) are good for saliva or swab collections. The solution can be applied on biosensor surfaces directly with good specificity. More studies are needed to characterize the effects of more complex samples, such as of patients with multiple infections and a mixture from multiple patients.

The biosensor surface must be functionalized to detect the nucleocapsid protein of SARS-CoV-2. All reagents were purchased from XLement of Wuhan, China. The gold-coated chip was immersed in 100 μM polycarboxylate solution (Cat. No. G40005) and incubated at 4 °C overnight, followed by three washes with ultra-pure water (UPW). Then the following steps were done at 37 °C. The chip was incubated with a 10 mM activation buffer (Cat. No. S20028) for 20 min. The SARS-CoV-2 nucleocapsid antibody (50 μg/mL, Cat. No. C10002) was immobilized on the biosensor surface for 3–4 h, followed by two washes with UPW. The antibody-immobilized biosensor was blocked for 30 min by block buffer (Cat. No. G30004), followed by treatment with a protective solution (Cat. No. G30006) for another 30 min. Then all liquids were removed to store biosensors for future use. Characterization of the binding layers is reported elsewhere [6]. Before use, the prepared biosensor chip was recovered in UPW. SARS-CoV-2 nucleocapsid protein (N protein, Cat. No. C10001) samples diluted with sample diluent (Cat. No. G30002) to 10 ng/μL were loaded on the biosensor surface. A cover glass was used to cover the liquid and flatten the liquid surface. The reflection spectrum was measured every 1 min in a 10-min process.

**Figure 5:**
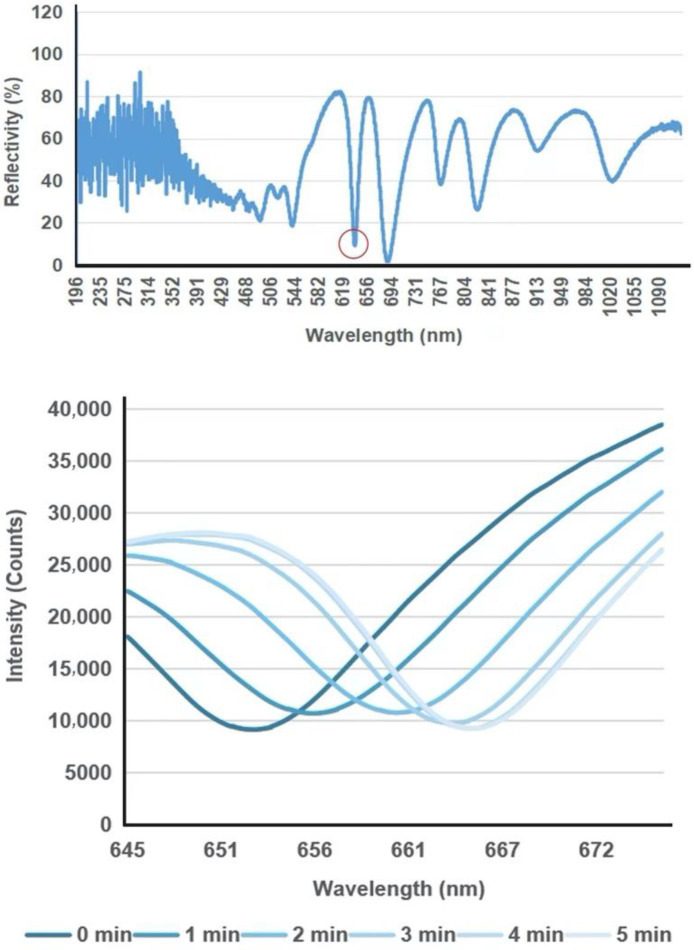
The biosensor’s response for detecting SARS-CoV-2 N protein (10 ng/μL). Above: characteristic valley of the reflection spectrum used for tracking the spectral shift is circled in red. Below: real-time shift of the characteristic valley as the N protein of SARS-CoV-2 is binding with the specific antibody.

Figure 5 shows representative spectral shifts of the biosensor upon binding reaction with the N protein of SARS-CoV-2 (10 ng/μL). Biosensors loaded with N protein samples were measured every 1 min; as the antibody captured the N protein on the biosensor surface, the characteristic valley of the reflection spectrum showed a continuous red shift change. Test sensitivity and specificity of the biosensor towards more complex samples such as swab samples of infected patients, the calibration curve of the biosensor and its comparison with PCR, and detection of S1 protein of SARS-CoV-2 with engineered T-ACE2 [23], are demonstrated in Supplementary Materials in back matter of this paper, and also reported elsewhere [6].

### 3.2. Photoacoustic Imaging

X-ray CT is the common imaging technique for human lung detection [24], which is an important complement to NAAT and gene sequencing methods for COVID-19. However, CT is a radiation method that is not safe for humans to use frequently. This technique cannot be used in a neonate due to the radiation and the instability of the detection because neonates are active and need to be fixed by adults. Therefore, photoacoustic imaging (PAI), a non-invasive imaging technique with high optical specificity and deep acoustic propagation depth, is expected to be widely used in COVID-19-related lung detection.

Ultrasonic sensors are the most important parts of PAI systems. We focus on designing high-performance ultrasonic transducers using integrated optical platform-based methods to detect photoacoustic signals. Based on the principle of the wearable photoacoustic imaging system, the structure of the photoacoustic probe is being designed. The pulsed light source is selected according to the property of the lung tissues. The photoacoustic signal receiver will be made and combined with the sensor designed based on the integration method. Then the controller system, data acquisition, and amplifier will be arranged considering the whole system structure and functions. The algorithms for data processing and image reconstruction will be conducted.

Before designing the whole wearable photoacoustic imaging system, the interaction between the optical and acoustic signals with lung tissues needs to be understood. Realizing the mechanism of the photoacoustic signal generation and propagation inside the lungs can prompt one to acquire high-quality photoacoustic signals and images. Therefore, we conducted a simulation model of photoacoustic imaging for lung tissues using COMSOL Multiphysics software to analyze the photoacoustic signal generation inside the lung tissues. The important processes of photoacoustic generation include optical propagation, optical absorption, and heat transfer.

**Figure 6:**
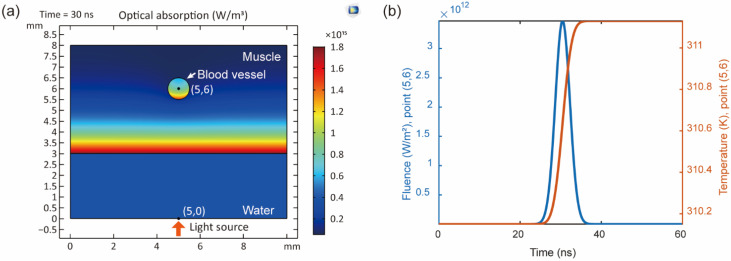
Simulation results of photoacoustic imaging for lung tissues: (**a**) Optical absorption at 30 ns, (**b**) Optical fluence and temperature change from 0–60 ns at points (5, 6).

Our simulation method followed the previous work [25]. Figure 6 shows the preliminary simulation results of optical absorption distribution, optical fluence, and temperature changes inside the simplified lung tissue model, including the muscle and blood vessels. The Gaussian pulsed light was input as an excitation source into the tissues through the water layer from point (5, 0). The optical absorption distribution at 30 ns is shown in Figure 6a. The optical absorption decreased with the depth increasing. At the same horizontal level, the blood vessel absorbs more light energy than the surrounding tissue, which provides the means to detect the blood vessel inside the tissue. If the data at points (5, 6) is extracted, the optical fluence and temperature changes as functions of time are shown in Figure 6b. When the optical fluence has an envelope of Gaussian function, the temperature increase gradually accumulates to generate a photoacoustic signal in one excitation.

The light energy reaches its maximum value when it is at the central time of one pulse at 30 ns. The temperature increases accumulatively following the excitation, which can represent a photoacoustic signal generation. Based on the simulation result, the potential of photoacoustic imaging for lung tissues is demonstrated.

### 3.3. Respiration Monitoring and Stimulation

Although currently, some sensing techniques may already have high sensitivity for detecting respiratory rate, long-term device stability and wearing comfort are still challenging. Therefore, it is necessary to concentrate on the rapid development of devices with long-term stability so that the accuracy of the device can be improved. In addition, searching for more appropriate materials to integrate breath sensors can improve wearing comfort while ensuring accuracy.

**Figure 7:**
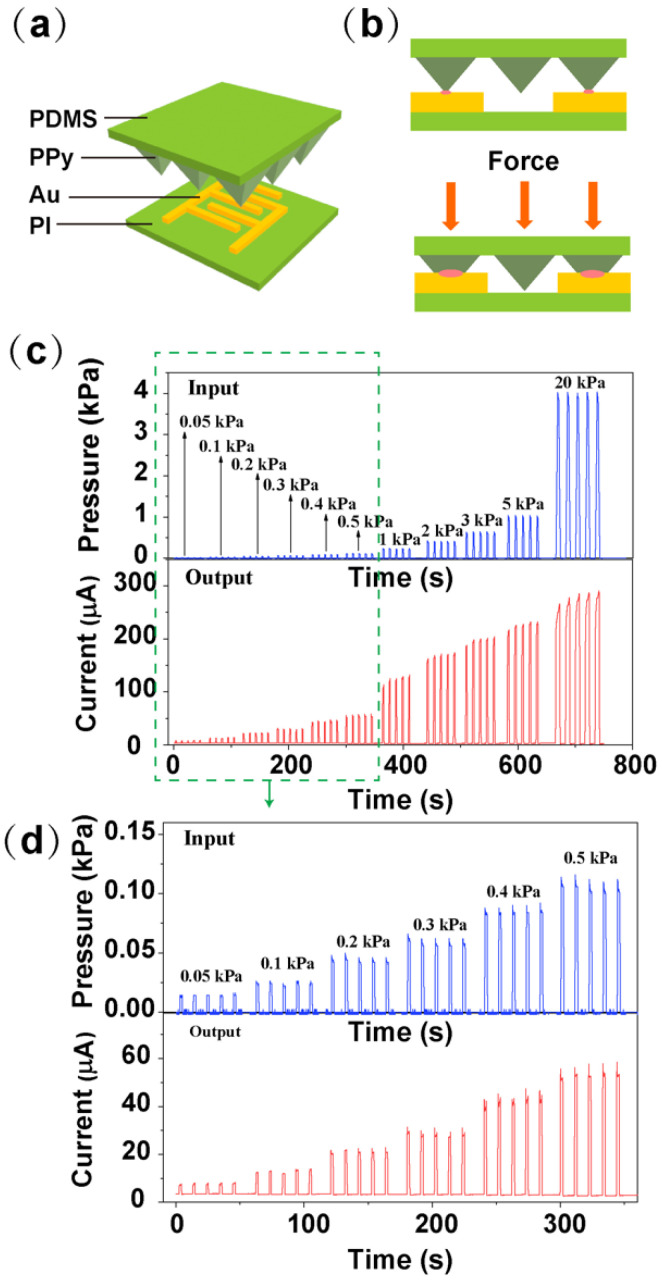
Results of the force sensor for respiration monitoring, (**a**,**b**) Working principle of the PPy-based force sensor, (**c**) Current-time curves of the sensor with different applied forces. (**d**) The magnified view of the current-time curves at applied force ranges from 0.05 kPa to 0.5 kPa.

The electrical stimulation system includes a respiratory monitor unit and electrical stimulation unit. As shown in Figure 7a, the respiratory monitor device is based on a micro-pyramidal resistive force sensor, which is composed of a polyimide (PI) substrate integrated with interdigitated Au electrodes and polydimethylsiloxane (PDMS) film with micro-pyramidal structure conformally coated with polypyrrole (PPy). The mechanism of the sensor is depicted in Figure 7b. When the force is applied to the sensor, PPy-based micro-pyramidal film is deformed, which leads to the increased contact area between interdigitated electrodes and PPy film, and therefore the resistance decreases. Figure 7c,d illustrates the instant current-time curve of the respiration sensor under repeated loading/unloading of different forces between 0.05 kPa to 20 kPa, which includes human respiration level.

**Figure 8:**
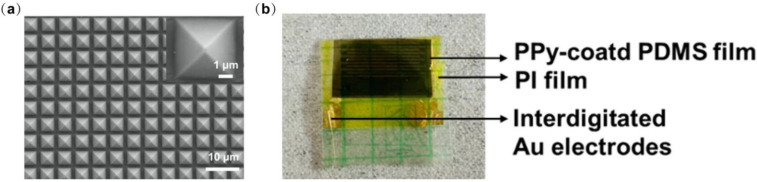
(**a**) SEM image of micro-pyramidal PPy-based PDMS surface. The inset illustrates a detailed SEM image of the individual structure. (**b**) Optical image of the device.

We conducted some characterization of the device. Figure 8a shows the SEM image of the micro-pyramidal PPy-based PDMS film. The optical image of the device layer is depicted in Figure 8b.

On the other hand, the electrical stimulation unit consists of two parts including communication and energy transfer through the inductive link. This link is built by two metal coils. One coil is placed outside the body. The other is embedded inside the body and under the skin. Generally, the power transfer efficiency of this inductive link is strongly related to the self-inductances of external coil L1 and internal coil L2, mutual inductance M, load resistance, and the operating frequency f. Here, the operating frequency is confined to 13.56 MHz to meet the industrial, scientific, and medical radio band requirements.

Here, we have simulated the magnetic field of an inductive coupling link through COMSOL Multiphysics. The electrical stimulation target is the hypoglossal nerve, above which the tissue is slim. We mimic this tissue thickness as a gap of around 6 mm, describing the distance between the external coil (Tx) and the internal coil (Rx). On the other hand, the diameter of the hypoglossal nerve is around 2.5 mm [26]. The internal coil would be placed upon this hypoglossal nerve, and its diameter is designed as 1cm, considering the needed power to be transferred for efficient stimulation of the hypoglossal nerve. The diameter of the Tx coil is defined as 5 cm.

**Figure 9:**
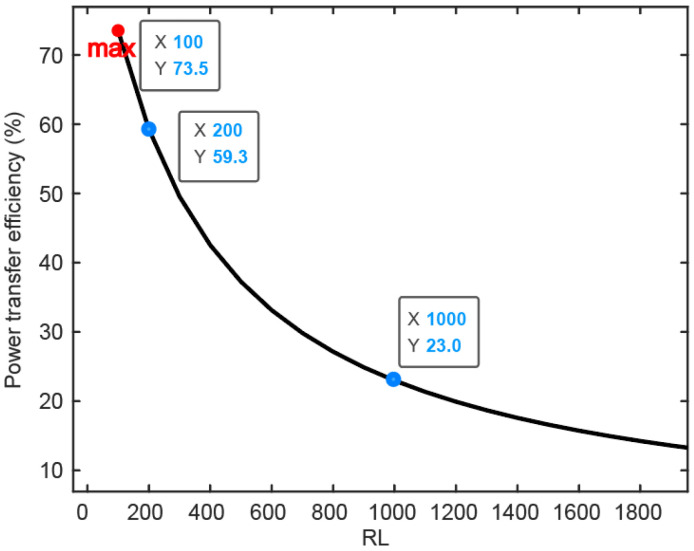
COMSOL-based simulation results of the magnetic field coupling link show the power transfer efficiency related to the load resistance.

The result of the simulation of the power transfer efficiency of the inductive link is depicted in Figure 9 in the function of the receiver overload. Through global evaluation, the self-inductances of the Tx and Rx are calculated as L1 = 16.057 uH and L2 = 0.887 uH, respectively. The mutual inductance between these two coils is M = 0.441 uH. As illustrated above in Figure 4, the energy received by the internal coil is input to the power management module (PMM), which means PMM works as a load connected to the inner coil. This load is named the RL in Figure 9. Based on the formula of PTE introduced in [27], PTE is strongly related to the value of RL. With process variation and transistor size, the RL varies from 100 Ω to several kΩ. While the impedance between stimulation electrodes is around 1 kΩ, the maximum value of RL is analyzed as 2 kΩ. With increased RL, power transfer efficiency (PTE) decreases significantly. When RL is 1 kΩ, PTE can be 23%. In terms of power consumption, the load between two stimulation electrodes consumes 4 mW if the stimulation current is 2 mA. As system-on-chip (SOC) embedded in the body consumes around 10% of the load power budget, the whole power consumption of the implantable part is 4.4 mW. Combined with the simulation results of PTE of the magnetic coupling link, the external power supply needs at least 22 mW.

## 4. Conclusions

The proposed closed-loop system includes several complementary topics. It consists of three subsystems. The wearable whole course surveillance, detection, and stimulation system are suitable for fighting COVID-19 pneumonia or other kinds of infectious diseases. The implementation of the proposed system is intended to produce a series of achievements with clinical application value, which will be used in epidemic diagnosis, treatment, prevention, and control. Moreover, the scientific research results produced by the project can also be widely used in possible future epidemics requiring rapid, low-cost detection, prevention and control, and treatment measures. The system provides a range of fast, high-throughput, non-invasive, portable, or wearable solutions to rapidly detect infected patients, curbing the rapid spread of current and future epidemics.

## Data Availability

The data presented in this study are available on request from the corresponding author. The data are not publicly available due to privacy restrictions.

## Supplementary Materials

The following supporting information can be downloaded at: https://www.mdpi.com/article/10.3390/bios12100900/s1, Figure S1. Detection of S1 protein of SARS-CoV-2. The resonant feature of the reflection spectrum shifts to longer wavelength as concentration of S1 protein increases. The shift saturates at about 0.2 nM of S1 protein concentration; Figure S2. Heigh profile of (a) PI film, (b) Au electrodes, (c) PDMS film and (d) PPy film measured by surface profiler, showing the thickness of 3.0 µm for PI film, 0.07 µm for Au electrodes, 5.2 µm for PDMS film and 0.8 µm for PPy film; Table S1. Test results analysis based on detection of various forms of specimens with the biosensing platform.

## Abbreviations

COVID-19: coronavirus disease 2019; CT: computed tomography; DBR: distributed Bragg reflector; LSPR: localized surface plasmon resonance; NAAT: nucleic acid amplification test; PAI: photoacoustic imaging; PCR: polymerase chain reaction; SARS-CoV-2: severe acute respiratory syndrome coronavirus 2; UTM: universal transport medium; UVM: universal viral medium; CDC: Center for Disease Control.
