# Supplementary material for: A Closed-Loop Approach to Fight Coronavirus: Early Detection and Subsequent Treatment
Source: Biosensors (Basel). 2022 Oct 20;12(10):900. doi: 10.3390/bios12100900 (PMC9599914; doi:10.3390/bios12100900)
Supplement: Supplementary file 1 [file biosensors-12-00900-s001.zip › biosensors-1901047-supplementary.pdf]

Supplementary Information

S1 protein of SARS-CoV-2 have been detected with the biosensing platform. The biosensors were first incubated in 11-mercaptoundecanoic acid (20 mM) in ethanol (10 ml) and incubated for 24 hours. The biosensors were then incubated in a mixture of 0.4 M 1-(3-dimethylaminopropyl)-3-ethylcarbodiimide hydrochloride (EDC) and 0.1 M N-hydroxy succinimide (NHS) in 2-(N-morpholino) ethane sulfonic acid (MES) buffer, for 15 minutes to activate the carboxylic groups of the biosensor surface. Engineered T-ACE2 (0.2 mg/ml) was immobilized on the biosensor surface for 30 minutes by covalent binding with activated carboxylic groups, followed by incubation in blocking buffer (Superblock, Sigma) containing bovine serum albumin (BSA) for 5 minutes in order to deactivate the unreacted carboxylic groups. Figure S1 shows the results of detection of different concentrations of S1 protein (Sino Biological) in phosphate buffer saline (PBS) buffer.

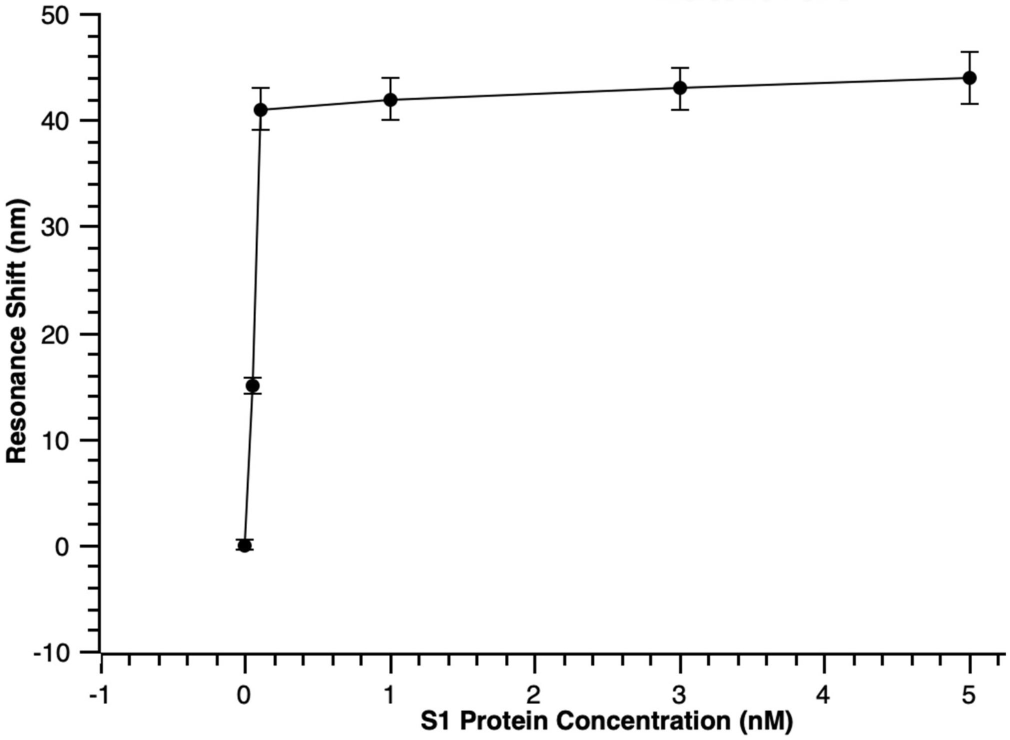

Figure S1. Detection of S1 protein of SARS-CoV-2. The resonant feature of the reflection spectrum shifts to longer wavelength as concentration of S1 protein increases. The shift saturates at about 0.2 nM of S1 protein concentration.

We have also carried out detection of various specimens with the novel biosensing platform. The various forms of specimens, the number of specimens, number of positive and negative specimens, as well as test sensitivity (or limit of detection), and test specificity, are listed in Table S1.

Table S1. Test results analysis based on detection of various forms of specimens with the biosensing platform.

Type of specimen	Number of specimens	Number of Positive Specimens	Number of Negative Specimens	Test sensitivity (S) or limit of detection (LoD)	Test specificity
Throat swab of diagnosed COVID-19 patients	24	24	0	LoD = 100 $TCID_{50}$/ml	
Thraot swab of patients infected by other pathogens	24	0	24	N/A	93%
Environmental specimens from inoculation sites	14	8	6	LoD ≅ PCR Ct value of 36	
S1 protein in buffer	24	24	0	S=200nm/(nM)	
N protein in buffer	24	24	0	S=50nm/(μg/ml)	

In addition, Figure S2 shows the thickness of each layer of the respiration monitoring device measured by surface profiler, demonstrating the thickness of ~3.0 μm for PI film, ~0.07 μm for Au electrodes, ~5.2 μm for PDMS film and ~0.8 μm for PPy film.

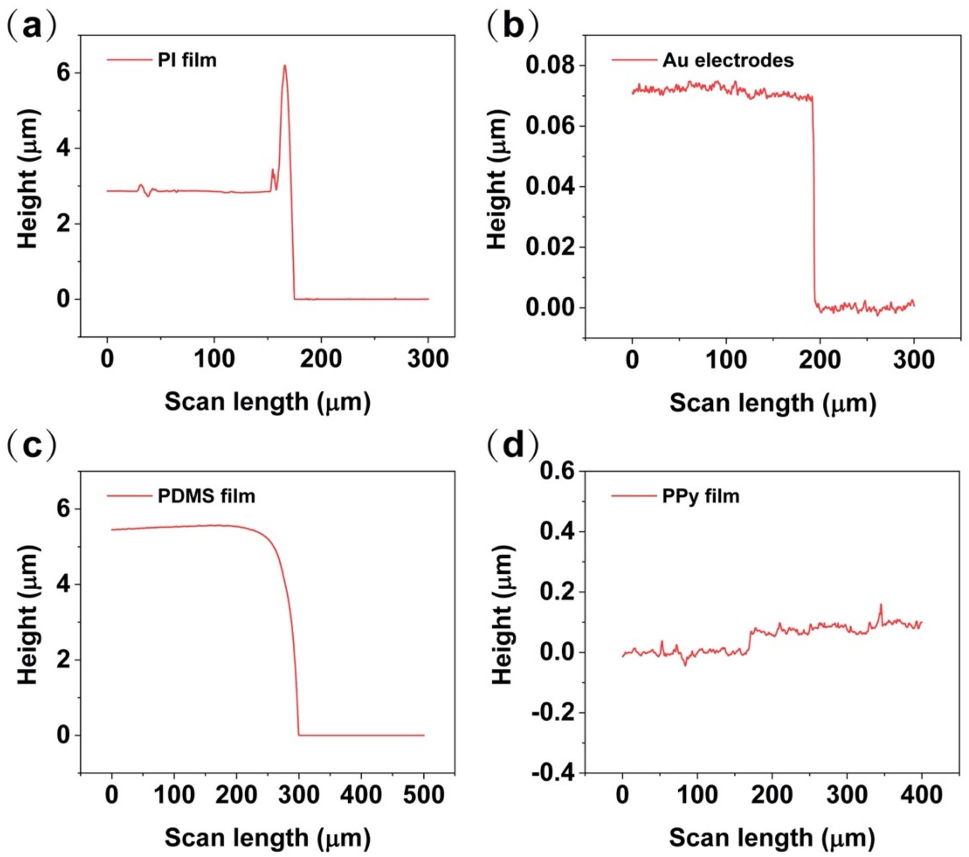

Figure S2. Heigh profile of (a) PI film, (b) Au electrodes, (c) PDMS film and (d) PPy film measured by surface profiler, showing the thickness of ~3.0 μm for PI film, ~0.07 μm for Au electrodes, ~5.2 μm for PDMS film and ~0.8 μm for PPy film.
